# Clonal Spreading of ST42 *Staphylococcus haemolyticus* Strains Occurs Possibly Due to *fusB* and *tetK* Resistant Genes and Capsule-Related Genes

**DOI:** 10.3390/ijms24076198

**Published:** 2023-03-24

**Authors:** Lee-Chung Lin, Shih-Cheng Chang, Yu-Hsiang Ou, Tsui-Ping Liu, Jang-Jih Lu

**Affiliations:** 1 Department of Laboratory Medicine, Chang Gung Memorial Hospital, Linkou, Taoyuan 333, Taiwan; 2 Department of Medical Biotechnology and Laboratory Science, College of Medicine, Chang Gung University, Taoyuan 33302, Taiwan; 3 Department of Medicine, College of Medicine, Chang Gung University, Taoyuan 33302, Taiwan

**Keywords:** *Staphylococcus haemolyticus* ST42, fusB, tetK, capsule related genes

## Abstract

Multi-drug resistant *Staphylococcus haemolyticus* is a frequent nosocomial invasive bacteremia pathogen in hospitals. Our previous analysis showed one of the predominant strains, ST42 originated from ST3, had only one multilocus sequence typing (MLST) variation among seven loci in *SH1431*; yet no significant differences in biofilm formation observed between ST42 and ST3, suggesting that other factors influence clonal lineage change. Whole genome sequencing was conducted on two isolates from ST42 and ST3 to find phenotypic and genotypic variations, and these variations were further validated in 140 clinical isolates. The fusidic acid- and tetracycline-resistant genes (*fusB* and *tetK*) were found only in CGMH-SH51 (ST42). Further investigation revealed consistent resistant genotypes in all isolates, with 46% and 70% of ST42 containing *fusB* and *tetK,* respectively. In contrast, only 23% and 4.2% ST3 contained these two genes, respectively. The phenotypic analysis also showed that ST42 isolates were highly resistant to fusidic acid (47%) and tetracycline (70%), compared with ST3 (23% and 4%, respectively). Along with drug-resistant genes, three capsule-related genes were found in higher percentage distributions in ST42 than in ST3 isolates. Our findings indicate that ST42 could become endemic in Taiwan, further constitutive surveillance is required to prevent the spread of this bacterium.

## 1. Introduction

Increasing opportunistic infections in neonates, older, and immunocompromised patients in hospital has led to the reclassification of coagulase-negative staphylococci (CoNS) as a threat [1,2,3,4]. Among all CoNS, *Staphylococcus haemolyticus* bacteria are considered the major staphylococci in nosocomial foreign device-related infections [5]. Moreover, the multi-drug resistant phenotype is common in *S*. *haemolyticus*, resulting in limitation and difficulty in treatment [6].

Most of the antibiotic resistance genes (ARGs) or virulence factors were thought to be spread via mobile genetic elements (MGEs) [7] across different staphylococci through horizontal gene transfer [7,8,9]. Several authors have performed whole-genome sequence (WGS) analyses to address this phenomenon in *S*. *haemolyticus* [10,11,12]. One study found that a commensal-origin group may adapt through MGEs in clinical environments [11]. However, these findings lack molecular typing information of the studied isolates, making it difficult to determine the impact of ARG and MGE on the epidemiological importance of those isolates. A recent similar study in China used isolates collected from feces, nares, and bronchoalveolar lavage fluid through a hospital ward environment. The results demonstrated that sequence type 42 (ST42) strains were more virulent than non-ST42 strains, making this the first study to identify the serious impact of the specific molecular type [12]. 

Our epidemiological surveillance and analysis of blood-isolated strains from burn patients revealed several novel STs, including the predominant type ST42 [4]. According to the eBurst analysis, ST42 originated from ST3, with only one variation among seven ST alleles. This variation allele (*SH1431* encoded *ebps*) was believed to be involved in the regulation of biofilm formation through Zn^2+^ binding [13,14]. In contrast, our biofilm formation results showed no significant difference between ST42 and ST3 strains. This suggests that other factors may lead to the prevalence of ST42 strains. The ST42 strain showed a gradual increase, whereas ST3 prevalence continually decreased after 2013. These trends represent a clonal lineage shift (Supplementary Figure S1). Although a previous comparative analysis revealed that ST42 is increasingly becoming a threatening *S*. *haemolyticus* type in China [12], none of their studied samples were blood-isolated strains. This indicates that there may be various explanations for our clonal lineage shift phenomena. To understand the mechanism through which this change occurs, we characterized both ST42 and ST3 clinical isolates. This characterization included WGS analyses of two isolates from each ST. 

Through the present study, we reveal that fusidic acid and tetracycline resistance may be a consequence of this clonal lineage change. Furthermore, the percentage of fusidic acid-resistant *S*. *haemolyticus* was insignificant in previous reports, but our data demonstrates high fusidic acid resistance rates in ST42, which is becoming a critical issue in hospital environments. In this study, we present the specific ARGs and virulence factors that may be responsible for ST42 prevalence in Taiwan.

## 2. Results

### 2.1. Comparative Analysis of Different Molecular Types of S. haemolyticus Genome Structures

**Figure 1:**
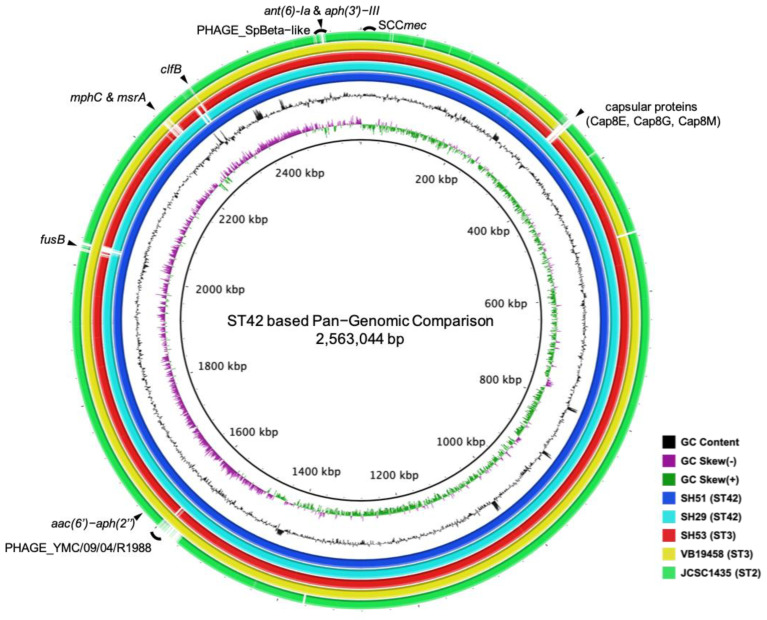
Comparative genomic analysis of genome structure of CGMH-SH51, CGMH-SH53, and the three reference strains.

To find the differences in genome compositions of ST3 and ST42 *S*. *haemolyticus*, whole genome sequences of CGMH-SH51 (ST42) and CGMH-SH53 (ST3) were compared with other ST3 and ST42 *S*. *haemolyticus* reference strains in GenBank (Figure 1 and Table 1). It depicts the comparative analysis of the genomic structures of our two strains (CGMH-SH51 and CGMH-SH53) and three reference strains (Figure 1, SH29, ST42; VB19458, ST3; and JCSC1435, ST2), which included important gene structures, MGEs (prophage and SCC*mec* cassette), ARGs, and virulence factors. All strains were isolated from blood, except for JCSC1435. Most of the characteristics, such as the number of coding sequences, transfer-messenger RNA (tmRNA), tRNA, rRNA, and GC content were similar across all compared strains. The GC-skew distribution showed an unsymmetrical pattern that is similar to other CoNS reported in a previous study [15]. Moreover, MGE analysis showed that all strains contained the SCC*mec* cassette and at least one prophage (Table 1). Prophage YMC/09/04/R1988 existed in four strains (both ST42 and ST3 strains). This prophage contains no drug-resistant genes and was identified as a lytic phage from methicillin-resistant *S*. *aureus* (MRSA) [16]. In contrast, a *ϕSPbeta*-like prophage containing two aminoglycoside resistant genes (*aph*(*3′*)-*III* and *ant*(*6*)-*Ia*) was found in CGMH-SH51 and CGMH-SH53 (Figure 1). Partial regions of the *ϕSPbeta*-like prophage were identified in SH29 and VB19458, which also contained *aph*(*3′*)-*III* and *ant*(*6*)-*Ia*.

The eight rings of the circular diagram (inner to outer) represent: GC content, GC skew (−) of CGMH-SH51, and GC skew (+) of CGMH-SH51. The dark blue ring represents the genome of CGMH-SH51 (ST42); the light blue ring represents the genome of SH29 (ST42, accession number CP011116.1); the red ring represents the genome of CGMH-SH53 (ST3); the yellow ring represents the genome of VB19458 (ST3, accession number CP045187); and the outer green ring represents the genome of JCSC1435 (ST2, accession number NC_007168). The black arcs represent SCC*mec*, *ϕSpbeta*-like prophage, and YMC/09/04/R1988 positions, whereas the black triangles indicate several drug-resistant or pathogenic factor genes.

**Figure 2:**
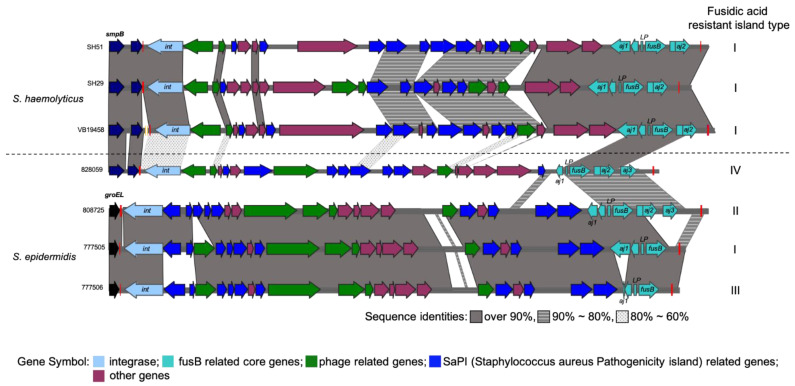
Pairwise comparison of the RI*_fusB_* structure.

Various ARGs, including macrolide, beta-lactam, and aminoglycoside were found in both ST3 and ST42 strains (Figure 1 and Table 1). Three macrolide-resistant genes were found in some strains (Table 2); *mphC*, *msrA* were identified in two of the ST42 strains and JCSC1435 whereas *ermC* was only present in the plasmid of JCSC1435 (pSHaeB, accession number AP006718). Furthermore, two beta-lactam-resistant genes (*blaZ* and *mecA*) were identified in all strains. Most of the *blaZ* were found in the *ϕSPbeta*-like prophage region. In contrast, the *blaZ* in JSCS1435 was found in a different region of the genome (Figure 1). D*frG*, a diaminopyrimidine-resistant gene, and three aminoglycoside modifying enzyme encoded genes (*aac*(*6′*)-*aph*(*2″*), *aph*(*3′*)-*III*, *and ant*(*6*)-*Ia*) were found in four strains. JCSC1435 only contained the *aac*(*6′*)-*aph*(*2″*) gene. Fusidic acid-resistant gene *fusB* was identified in both ST42 strains and in one ST3 strain (CGMH-SH51, SH29, and VB19458; Table 2). This gene is located in the fusidic acid-resistant island (RI*_fusB_*) (Figure 2). All three RI*_fusB_* belong to type I RI*_fusB_*. In addition, they are all located downstream of *smpB* and share high sequence similarities in partial regions, especially in the *fusB*-related core genes region (Figure 2). We also compared fusidic acid-resistant islands in *S*. *epidermidis* and found that most of the SeRI*_fusb_* were different from ShRI*_fusb_*, with the exception of SeRI*_fusB_*_-828059_. Locations of most SeRI*_fusb_* were downstream of *groEL* and their structures were highly conserved. Unlike the other SeRI*_fusb_*, SeRI*_fusB_*_-828059_ has the same position as ShRI*_fusb_*, and its sequence is located near the *smpB* region. 

The structural similarities of seven fusidic acid-resistant islands located on either three *S. haemolyticus* or four *S. epidermidis* strains. All fusidic acid-resistant islands located on *S. haemolyticus* were inserted near *smpB* and most of those located on *S. epidermidis* were inserted near *groEL.* Only 828059 was inserted near *smpB*. These islands were classified into different types based on a previous report [17] and are labeled on the left-hand side of this figure. Sequence similarities are shaded using different gray scale. >90% identities are presented by dark gray, 90%~80% similarities are presented by middle gray, and 80%~60% similarities are presented by pale gray. Different genes and their symbols are represented by various colors. The dark blue followed by *smpB* is *ssrA*, a tmRNA that is considered a target for MGE transfer. The yellow brackets in *S. haemolyticus* VB19458 indicate a 40,000 length flanked region, which was not similar to any sequence of the fusidic acid-resistant islands located in the other six strains.

**Figure 3:**
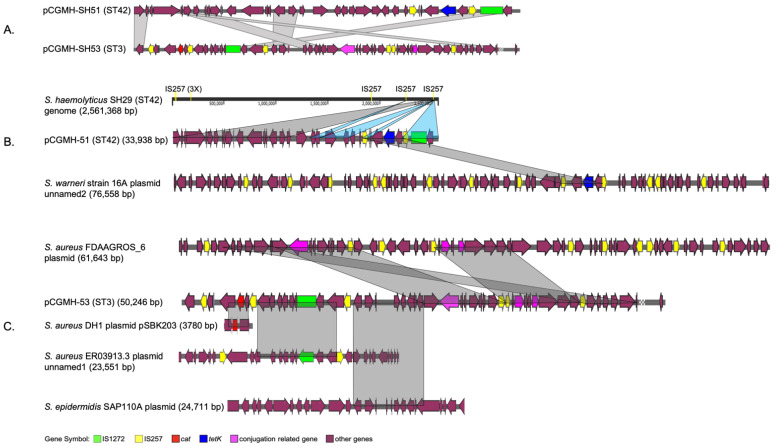
Pairwise comparison of CGMH-SH51 and CGMH-SH53 plasmids and their most identical sequences. Plasmids of CGMH-SH51 (pCGMH-SH51) and CGMH-SH53 (pCGMH-SH53) were compared with each other (**A**) and their most identical sequences, as identified from NCBI blast, are shown in (**B**,**C**). Grayscale indicates sequence similarities > 90%. Light blue indicates sequences close to the CGMH-SH51 genome (similarity > 90%). Gene Symbols and different genes are represented by various colors.

Three strains (CGMH-SH51, CGMH-SH53, and JCSC1435) contained plasmids, with 44% similarity between the CGMH-SH51 and CGMH-SH53 plasmids (pCGMH-SH51 and pCGMH-SH53, Figure 3A). Sequence blast analysis revealed that both plasmids in CGMH-SH51 and CGMH-SH53 comprised of multiple fragments from various origins (Figure 3B,C). Most pCGMH-SH51 regions came from the SH29 genome (73%) and a few regions (15%) came from the *S. warneri* strain 16A plasmid unnamed2 (CP031268) (Figure 3B). Partial regions identical to the SH29 genome are also close to the CGMH-SH51 genome (Figure 3B). Four varying origins made up most of the pCGMH-SH53 structure (Figure 3C). We determined that 47% of pCGMH-SH53 regions came from the *S. aureus* FDAAGROS_6 plasmid, whereas the other regions originated from *S. aureus* strain ER03913.3 plasmid unnamed1 (CP030482; 16.8%), *S. epidermidis* plasmid SAP110A (GQ900465; 14.7%), and *S. aureus* strain DH1 plasmid pSBK203 (SAU35036; 3.9%). Both pCGMH-SH51 and pCGMH-SH53 contained ARGs. T*etK,* a tetracycline-resistant gene was found in pCGMH-SH51. Similarly, a chloramphenicol-resistant gene (*cat*) was found in pCGMH-SH53. Most of the aforementioned regions were flanked by IS257. In addition to the ARG, a conjugation-related gene was identified in the CGMH-SH53 plasmid. We isolated this plasmid to process the conjugation with the ST42 strain. However, we failed to get the conjugants. 

### 2.2. Distribution of Antibiotic-Resistant Genotypes and Phenotypes among the Collected ST3 and ST42 Clinical Isolates

To understand the genotypic and phenotypic variation of the ARGs between 48 ST3 and 92 ST42 isolates, we further analyzed the distribution of these ARGs and performed an antimicrobial phenotypic test on our clinical isolates (Table 2). For fusidic acid resistance, we found that genotypic distribution of *fusB* and the fusidic acid-resistant phenotype were significantly higher in ST42 (46.7%, 45.6%) than in ST3 isolates (22.9%, 22.9%) (Table 2). All 53 fusidic acid-resistant isolates were verified as containing type I ShRI*_fusB_*. Further investigation of these isolates revealed that MICs of most isolates were 8 μg/mL. Moreover, five of the ST42 isolates had even higher MIC at 16 μg/mL (Table 3). 

The lincosamide- and macrolide-resistant phenotypes were similar between CGMH-SH51 (ST42) and CGMH-SH53 (ST3). Approximately 70% of both populations were resistant to clindamycin and all isolates were resistant to erythromycin. The genotypic distribution of *ermC* was similar in both ST42 and ST3 strains (87% and 87.5%). Additionally, *mphC* and *msrA* contents were higher in ST42 (both genes were 99%) than in ST3 (both genes were 75%). We also investigated the distribution of *ermA*, which was not detected in any of the isolates. The distribution of *tetK* and *cat* genes showed contrasting results. Our data showed that the distribution of *tetK* and the tetracycline-resistant phenotype were considerably higher in ST42 than in ST3 (70.1% versus 4.2%, Table 3). In contrast, the distribution of *cat* and the chloramphenicol-resistant phenotype was higher in ST3 than in ST42 (14.6% versus 4.3%). Further investigation of the co-existence of multiple ARGs was conducted through cross comparison of the *fusB* and *tetK* positive isolates. This investigation indicated that nearly 46% of ST42 isolates (42 from 92 ST42 isolates) contained both *fusB* and *tetK*. However, none of the ST3 isolates simultaneously contained both genes (Supplementary Table S1). Due to the concern about the correlation between drug usages and bacterial resistant rates, we analyzed the topic usage amount of fusidic acid and tetracycline in Linkou Chang Gung Memorial Hospitals from 2011 to 2017 with both drug resistant proportion rates (Supplementary Figure S2), the results showed no significant correlation between them. 

### 2.3. Distribution of Virulence Factor Genes between ST3 and ST42 S. haemolyticus 

Several virulence factors were found in both ST42 strains (Figure 1). These included three capsule formation-related genes (*Cap8E*, *Cap8G*, and *Cap8M*) and *ClfB*, which are considered responsible for bacterial aggregation and adhesion during infection [18]. Here, we performed a surveillance of these virulent factors in our collected clinical isolates and found that these four virulence factor genes existed in higher percentages in ST42 than in ST3 (Table 2). *Cap8E* and *cap8G* distribution was 87% and 25%, respectively. Similarly, *cap8M* distribution was 86% versus 15% whereas *clfB* was 64% versus 2%.

## 3. Discussion

Epidemiological surveillance showed that *S*. *haemolyticus* ST3 was the original strain that evolved into many other molecular types. However, ST42 has become the predominant lineage in Taiwan [4]. We previously showed that ST42 and ST3 possess only one MLST locus variation among seven loci in SH1431, which has been reportedly involved in the regulation of biofilm formation through Zn^2+^ binding affinities. However, biofilm formation assay revealed no significant differences between ST3 and ST42 [4]. Moreover, the present study showed that multiple drug-resistant genes and virulence factors in ST42 may play important roles in this manner.

Comparative genomic structure analysis showed that both of our WGS strains contain the SCC*mec* cassette (Figure 1 and Table 1), which is consistent with previous observations that the oxacillin-resistant phenotype was common in *S*. *haemolyticus* [19,20,21]. In addition to the SCC*mec* cassette, our study showed that several ARGs in CGMH-ST51 (ST42) and CGMH-ST53 (ST3) were located inside the *ϕSPbeta*-like prophage, which has been reported to contain multiple antibiotic-resistant genes [22,23]. Although some ARGs identified in the present study were not located inside the MGE, previous studies showed that *mphC*, *msrA*, and *aac*(*6′*)-*aph*(*2″*) were encoded by plasmids or transposons [7]. This suggests that these ARGs may have been transferred by MGEs into the *S*. *haemolyticus* genome in the past. It may be a general scheme in *S*. *haemolyticus* and may have resulted in the multiple drug-resistant phenotype in *S*. *haemolyticus* [1,21].

Here, ST42 contained most of the drug-resistant genes, which suggests that ST42 may reflect an evolutionary trend and utilize these multiple drug-resistant genes to compete with the strains. According to the eBurst analysis in our previous studies, ST3 was the founder molecular type, whereas ST2 and ST42 evolved from and extended through ST3 [4]. This study suggests that several ARGs may be transferred by MGE into ST42, leading to the prevalence of this group in ward environments.

Our plasmid structure comparison could provide evidence that the transfer of ARGs might be mediated by MGEs. Both structures of pCGMH-SH51 and pCGMH-SH53 comprised of multiple fragments, most of which originated from various plasmids. These fragments, including the ARGs on both plasmids, were flanked by IS257 in their originated plasmid. IS257 is associated with the tetracycline-resistant gene (*tetK*) [7], which was identified in the CGMH-SH51 plasmid. Although chloramphenicol resistance has been associated with IS26, the *cat* gene in the CGMH-SH53 plasmid was flanked by IS257, which also belongs to the IS26 family [24]. These observations strongly suggested that the clonal drug-resistant genes may be mediated by MGEs.

Fusidic acid is a protein synthesis inhibitor, which interact with elongation factor G (EF-G) to prevent translation continuing [25]. It rarely has cross-resistance with other groups antibiotics and lower side effect, which made it widely used as topical antibiotic for most staphylococci treatment, such as skin or prosthetic joint infection [26]. However, due to the unrestricted commonly usage, which increasing the bacterial resistance [26]. Previously studies showed fusidic acid resistant are either found in *fusA* mutation or mobile element mediated resistant genes [27], and *fusB* is one of the common prevent fusidic acid resistant gene among staphylococci [28]. The *fusB*-containing resistant island is responsible for the fusidic acid-resistant phenotype [28,29,30],which has been found in low proportions (20%) in CoNS [31]. Compared to previous studies, our results showed a similar resistant rate in ST3 (22.9%, Table 2) and approximately twice the resistant rate in ST42 (46.7%, Table 2). These results suggest that ST42 may take advantage of this resistance for clonal spreading. Previous studies in *S*. *epidermidis* showed that *aj1* may play a critical role in fusidic acid resistance strength [17]. SeRI*_fusB_* with partially truncated (type II) or full-length *aj1* (type I) in *aj1*-LP-*fusB* fragments expressed higher fusidic acid resistance. In addition, most of their MIC were 16 μg/mL or higher [17]. All our ShRI*_fusB_* contained full-length *aj1*. However, their MIC was not as high as that of SeRI*_fusB_*, which indicates that RI*_fusB_* may express varying resistance strength in different species. 

The cross comparison of *fusB* and *tetK* positive isolates showed that only ST42 isolates simultaneously contained both genes. This suggests that it may be easier for ST42 to become a multiple-ARG strain than ST3. Recent comprehensive studies of *S*. *haemolyticus* from various sources in China showed that ST42 isolates have a higher tetracycline resistant rate and higher proportion of *fusB* [12]. These results indicate that multiple ARGs may commonly exist in ST42 isolates.

The genotypic distribution of *mphC* and *msrA* were higher in ST42 than in ST3. However, the macrolide resistant phenotypic distribution was similar in ST42 and ST3, which suggests that the other macrolide resistant genes may be responsible for it. The *erm* family plays an important role in the macrolide resistant mechanism, especially for *ermA* and *ermC*, which are major *erm* family genes found in *staphylococci* [32,33]. Although *ermA* was not found in our isolates, the high proportion of *ermC* distribution in both ST3 and ST42 isolates may contribute to the similar resistant phenotypic distribution in both ST3 and ST42 populations.

Capsular polysaccharides have been identified as responsible for the virulence of bacterial strains [34,35]. The function of these polysaccharides in *S*. *aureus* may be to protect them against phagocytosis during the pathogenesis [36,37]. Here, three of the capsule synthesis-related genes were found in most of the ST42 isolates. This could lead to a postulation that those *cap* genes are responsible for the formation of certain capsular polysaccharides and provide protection to against the host immune response. Recent comprehensive studies of *S*. *haemolyticus* in China also found several *cap* genes in ST42, and their animal model demonstrated that ST42 strains were more virulent than non-ST42 isolates [12]. In addition to the capsular polysaccharides, clumping factor B (*clfB*) is involved in fibrinogen adhesion [38], which is critical for bacterial pathogenesis and infection [39]. Over half of our clinical ST42 isolates contained this virulence factor, and only one ST3 isolate has it. This is further evidence that ST42 may be more virulent than ST3. 

There are some limitations in this study. First, although clinical isolates were collected from two different medical centers located in North and South Taiwan, more isolation sources (i.e., different medical centers) may be helpful to represent geological diversities. Second, the greater similarities between the pCGMH-SH51 and SH29 genomes than that of CGMH-SH51 suggested that pCGMH-SH51 may be more closely related to SH29. However, previous studies of SH29 have not mentioned the similar, plasmid-like pCGMH-SH51, the origin of which is difficult to distinguish between SH29 and CGMH-SH51 and its plasmid. Further analysis is necessary to resolve this question. Last, this study lacked an animal model to elucidate the impact of the three capsule synthesis-related genes on strain virulence. Nevertheless, the importance of capsule synthesis-related genes has been reported in a previous study [12] which supports our conjecture. 

In conclusion, our study revealed two drug-resistant genes, namely *fusB* and *tetK*. These genes have a much higher preference rate in ST42 than in ST3, which provide more disadvantages during drug treatment. Since low proportion of resistant isolates in previous report, little has been mentioned about clinical fusidic acid treatment failure [26], which has highlighted importance of our finding that detection of these resistant genes is precaution and suggesting for clinical treatment combination with multi-antimicrobial drugs in future. Furthermore, capsule synthesis-related genes and adhesion factor may also protect ST42 isolates against eradication. All the aforementioned genes were responsive for the survival competition under stress conditions and may be the reason for the dominance of ST42.

## 4. Materials and Methods

### 4.1. Bacterial Isolates

A total of 140 *S*. *haemolyticus* isolates were collected from the Chang Gung Memorial Hospitals in Linkou and Kaohsiung between 2010 and 2017 [3,4]. The two medical centers are located in northern and southern Taiwan, respectively. All strains were isolated from blood specimens and molecular typed using multilocus sequence typing (MLST), as previously described [40]. Additionally, all isolates were methicillin resistant. Ninety-two isolates belonged to ST42 and forty-eight isolates belonged to ST3. One of the ST42 (CGMH-SH51) and ST3 (CGMH-SH53) isolates were selected for WGS analysis. The detailed information of all strains is listed in Supplementary Figure S1 and Supplementary Table S1. 

### 4.2. Whole-Genome Sequencing and Annotation

*S*. *haemolyticus* strains CGMH-SH51 and CGMH-SH53 were grown on tryptic soy broth (TSB) medium overnight for genomic DNA extraction and further analysis. The PacBio™ method (Pacific Biosciences, Menlo Park, CA, USA) was utilized to analyze the whole-genome sequences of these two strains. Further genome assembly was completed as follows: Flye, a de novo assembler [41] was used for contig assembly; contigs scaffolding was applied using SSPACE [42]; and scaffolds were finally polished using Arrow algorithm (https://github.com/PacificBiosciences/GenomicConsensus, accessed on 15 February 2023). Gene annotation was generated using Prokka v1.12 (https://github.com/tseemann/prokka/, accessed on 15 February 2023), which is designed for bacterial or viral genome annotation. Next, the quality of the assembled genome was evaluated using Quast v4.5 [43]. The annotated data were further verified using the RAST web annotation service (Rapid Annotation using Subsystem Technology, https://rast.nmpdr.org/, accessed on 15 February 2023) to determine the function of each gene. The BLAST Ring Image Generator (BRIG, http://brig.sourceforge.net/, accessed on 15 February 2023) was used for visualization of the circular genome and comparative genomic analysis of individual strains. Plasmid sequence similarities were processed using Artemis (http://sanger-pathogens.github.io/Artemis/Artemis/, accessed on 15 February 2023) and BLAST service from NCBI (National Center for Biotechnology Information; https://blast.ncbi.nlm.nih.gov/Blast.cgi, accessed on 15 February 2023). The prophage search was performed using “PHAST” (PHAge Search Tool, http://phast.wishartlab.com/index.html, accessed on 15 February 2023) and “PHASTER” analyses (http://phaster.ca/, accessed on 15 February 2023). Moreover, virulence factors were identified using the Virulence Factors of Pathogenic Bacteria Database (VFDB, http://www.mgc.ac.cn/VFs/main.htm, accessed on 15 February 2023) web service, whereas antimicrobial resistance genes were analyzed using the Comprehensive Antibiotic Resistance Database (CARD, https://card.mcmaster.ca/, accessed on 15 February 2023). All the above information was organized through the service of the Bacterial and Viral Bioinformatics Resource Center (BV-BRC^3.25.3^; https://www.bv-brc.org/, accessed on 15 February 2023). Additionally, CGMH SH51 and CGMH SH53 have been submitted to GenBank as BioProject PRJNA781382. Two Bio Samples, namely SAMN23247097 and SAMN23247098, represent the genome and plasmid sequences of CGMH-SH51 and CGMG-SH53, respectively. SH29 (accession number CP011116), VB19458 (accession number CP045187), and JCSC1435 (accession number AP006716) were reference strains obtained from the NCBI GenBank. 

### 4.3. Antimicrobial Susceptibilities Assay

The antimicrobial phenotype was characterized using the disk diffusion method, as outlined in the CLSI guidelines [44]. Briefly, fusidic acid, clindamycin, erythromycin, and tetracycline disks were placed on the surface of the bacterium-grown medium. Next, their susceptibilities were evaluated via the inhibition zone. The minimal inhibition concentration (MIC) determination method of fusidic acid was performed as previously described [17].

### 4.4. Drug-Resistant Genotype and Virulence Factors Characterization

Drug-resistant genes and virulence factors among the collected isolates were examined using PCR. The relative primers used in these PCR reactions are listed in Table 4. The PCR conditions detecting drug-resistant genes (including *fusB*, *ermA*, *ermC*, and *tetK*) were set up as previously described [31,45,46,47]. However, the *mphC*, *msrA*, and *cat* detection were designed in this study. The PCR conditions for *cat* and *msrA* were: 98 °C for 10 seconds; 30 cycles of 98 °C for 5 seconds; 55 °C for 5 seconds; 72 °C for 20 seconds; and a final extension of 72 °C for 10 min. The PCR conditions for *mphC* were similar to those for *msrA*, with the exception of the annealing temperature (changed to 58 °C). Types of fusidic acid resistance islands were based on the *aj1*-LP-*fusB* fragment and defined as previously described [17,48]. In brief, complete *aj1* in the *aj1*-LP-*fusB* fragment was classified as type I; *aj1* with partially deleted 5′ regions was type II; and the mostly truncated *aj1* left with only a few 5´ regions was considered as type III. PCR primer sets *aj1* 606-577R and *fusB* 389-361R were used for the detection of the *aj1*-LP-*fusB* fragment, and all products were sequenced. PCR conditions for detecting virulence factors were designed in this study and were as follows: 98 °C for 10 seconds; 30 cycles of 98 °C for 5 seconds; 54 °C for 5 seconds; 72 °C for 20 seconds; and a final extension of 72 °C for 10 min.

## Figures and Tables

**Table 1:** General information of WGS analysis of five *S. haemolyticus* strains.

Strain	CGMH_SH51	SH 29	CGMH_SH53	VB19458	JCSC1435
MLST	ST42	ST42	ST3	ST3	ST2
Size (bp)	2,563,044	2,561368	2,586,626	2,699,210	2,685,015
Clinical origin	blood	blood	blood	blood	unknown
Number of CDS	2618	2456	2593	2568	2678
tmRNA	1	1	1	1	1
tRNA	61	60	63	60	59
rRNA	19	16	16	19	16
G+C content	33%	33%	33%	33%	33%
SCC*mec*	+	+	+	+	+
Plasmid	1	0	1	0	3
Prophage	PHAGE_Staphy_YMC/09/04/R1988_NC_022758 (1559323–1600746 bp)	PHAGE_Staphy_YMC/09/04/R1988_NC_022758 (1,552,815–1,609,358 bp)	PHAGE_Staphy_YMC/09/04/R1988_NC_022758 (1,584,557–1,625,922 bp)	PHAGE_Staphy_YMC/09/04/R1988_NC_022758 (1,652,339–1,698,371 bp)	PHAGE_Staphy_CNPx_NC_031241 (2,346,384–2,410,301 bp)
	PHAGE_Staphy_SPbeta_like_NC_029119 (2,502,492–2,527,281 bp)		PHAGE_Staphy_SPbeta_like_NC_029119 (2,464,491–2,514,683 bp)	Staphy_IME_SA4_NC_029025 (2,125,445–2,197,547 bp)	
**ARGs (Antimicrobial Resistant Genes)**					
*mphC*	+	+	-	-	+
*msr(A)*	+	+	-	-	+
*blaZ*	+ (prophage)	+ (prophage)	+ (prophage)	+ (prophage)	+
*mecA*	+ (SCC*mec*)	+ (SCC*mec*)	+ (SCC*mec*)	+ (SCC*mec*)	+ (SCC*mec*)
*aac(6′)-aph(2″)*	+	+	+	+	+
*aph(3′)-III*	+ (prophage)	+ (prophage)	+ (prophage)	+ (prophage)	-
*ant(6)-Ia*	+ (prophage)	+ (prophage)	+ (prophage)	+ (prophage)	-
*dfrG*	+	+	+	+	-
*fusB*	+	+	-	+	-
*tetK*	plasmid	-	-	-	-
*cat*	-	-	plasmid	-	-
*ermC*	-	-	-	-	plasmid
**Virulence Factors**					
Cap8E	+	+	-	-	+
Cap8G	+	+	-	-	+
Cap8M	+	+	-	-	-
ClfB	+	+	-	-	-

+: indicated in the chromosome.

**Table 2:** Distribution of antibiotic-resistant genes and virulence factors among clinical collected 92 ST42 and 48 ST3 strains.

		ST42 [n, (%)]		ST3 [n, (%)]	
Antibiotics	Resistant Gene	Phenotypic Distribution	Genotypic Distribution	Phenotypic Distribution	Genotypic Distribution
Fusidic acid	*fusB*	43 (46.7)	42 (45.6)	11 (22.9)	11 (22.9)
Tetracycline	*Tet(A)*	65 (70.1)	65 (70.1)	2 (4.2)	2 (4.2)
Chloramphenicol	*cat*	4 (4.3)	4 (4.3)	7 (14.6)	7 (14.6)
Clindamycin Erythromycin	*mph(C)* *msrA*	56 (60.8) 92 (100)	91 (99)	32 (66.7) 48 (100)	36 (75)
	*ermC*		80 (87)		42 (87.5)
**Virulence Factors**		**Prevalence of Virulence Factors**		**Prevalence of Virulence Factors**	
Cap8E		87 (94.6)		25 (59.5)	
Cap8G		87 (94.6)		25 (59.5)	
Cap8M		86 (93.5)		15 (35.7)	
ClfB		64 (69.6)		2 (4.8)	

**Table 3:** Distribution of fusidic acid MICs among 53 *S. haemolyticus* fusidic acid resistant isolates.

MSLT	MIC (μg/mL)	No (%)
3	4	3 (27.3%)
	8	8 (72.7%)
42	4	1 (2.3%)
	8	36 (83.7%)
	16	5 (14%)

**Table 4:** Primers used in the experiments.

PCR Targets	Primer Name	Oligo Sequence (5′-3′)	Reference
*fusB*	FusB-F	TCATATAGATGACGATATTG	[31]
	FusB-R	ACAATGAATGCTATCTCGAC	
*aj1*-LP-*fusB*	aj1 606-577R	AGTAAAGAATAAGTTTTTAATCGTTAATGC	[17]
	*fusB* 389-361R	TTCCGATTTGATGCAAGTTCATTCCATCC	
*mphC*	*mphC*-F	GAGACTACCAAGAAGACCTGACG	this study
	*mphC*-R	CATACGCCGATTCTCCTGAT	
*msrA*	*msrA*-F	CCTATGCATACAACCGACAG	
	*msrA*-R	CTACACCATTTGCACCTACG	
*ermA*	*ermA*-F	GTTCAAGAACAATCAATACAGAG	[46]
	*ermA*-R	GGATCAGGAAAAGGACATTTTAC	
*ermC*	*ermC*-F	GGTGTAATTTCGTAACTGCC	[47]
	*ermC*-R	TAATGCCAATGAGCGTTTTG	
*tetK*	*tet*-F	TCGATAGGAACAGCAGTA	[45]
	*tet*-R	CAGCAGATCCTACTCCTT	
*cat*	*cat*-F	TGGTAACCATCACATACCGCA	this study
	*cat*-R	GTGAGGGAAATTTGGGTTATTG	
*cap8E*	*cap8E*-F	CTTTAACGGTGACAGATCCA	this study
	*cap8E*-R	CACACTGTGCATACTCTTCT	
*cap8G*	*cap8G*-F	TACTTAGAAGCAGTTGGCAG	this study
	*cap8G*-R	TTCTTCGGGTACATTTTGGT	
*cap8M*	*cap8M*-F	ACTCATAGTAGCTGGACCTT	this study
	*cap8M*-R	CCCATAACTTGAGCTAGTCC	
*clfB*	*clfB*-F	TTTTGAGGGTTGGATAACTGA	this study
	*clfB*-R	TCTGCAGAACCATTACCTTT	

## Data Availability

Data are available at request from the authors.

## Supplementary Materials

The following supporting information can be downloaded at: https://www.mdpi.com/article/10.3390/ijms24076198/s1.

## References

[1] Silva P.V., Cruz R.S., Keim L.S., Paula G.R., Carvalho B.T., Coelho L.R., Carvalho M.C., Rosa J.M., Figueiredo A.M., Teixeira L.A. (2013). The antimicrobial susceptibility, biofilm formation and geno-typic profiles of staphylococcus haemolyticus from bloodstream infections. Mem. Instig. Oswaldo Cruz.

[2] Teeraputon S., Santanirand P., Wongchai T., Songjang W., Lapsomthob N., Jaikrasun D., Toonkaew S., Tophon P. (2017). Prevalence of methicillin resistance and macrolide-lincosamide-streptogramin B resistance in Staphylococcus haemolyticus among clinical strains at a tertiary-care hospital in Thailand. New Microbes New Infect..

[3] Chang P.H., Liu T.P., Huang P.Y., Lin S.Y., Lin J.F., Yeh C.F., Chang S.C., Wu T.S., Lu J.J. (2018). Clinical features, outcomes, and molecular characteristics of an outbreak of staphylococcus haemo-lyticus infection, among a mass-burn casualty patient group, in a tertiary center in northern Taiwan. J. Microbiol. Immunol. Infect.

[4] Lin L.-C., Liu T.-P., Chang S.-C., Lu J.-J. (2022). Characterization of new *Staphylococcus haemolyticus* ST42 populations in northern Taiwan. Microb. Drug Resist..

[5] Fredheim E.G.A., Klingenberg C., Rohde H., Frankenberger S., Gaustad P., Flægstad T., Sollid J.E. (2009). Biofilm formation by *Staphylococcus haemolyticus*. J. Clin. Microbiol..

[6] Czekaj T., Ciszewski M., Szewczyk E.M. (2015). Staphylococcus haemolyticus—An emerging threat in the twilight of the antibiotics age. Microbiology.

[7] Partridge S.R., Kwong S.M., Firth N., Jensen S.O. (2018). Mobile genetic elements associated with anti-microbial resistance. Clin. Microbiol. Rev..

[8] Becker K., Heilmann C., Peters G. (2014). Coagulase-negative staphylococci. Clin. Microbiol. Rev..

[9] Argemi X., Hansmann Y., Prola K., Prévost G. (2019). Coagulase-negative staphylococci pathogenomics. Int. J. Mol. Sci..

[10] Furi L., Haigh R., Al Jabri Z.J., Morrissey I., Ou H.Y., Leon-Sampedro R., Martinez J.L., Coque T.M., Oggioni M.R. (2016). Dissemination of novel antimicrobial resistance mechanisms through the insertion sequence mediated spread of metabolic genes. Front. Microbiol..

[11] Maria P., Erik H., Claus K., Jorunn P.C. (2019). Comparative genomic analysis of staphylococcus haemo-lyticus reveals key to hospital adaptation and pathogenicity. Front. Microbiol..

[12] Qin M., Chen P., Deng B., He R., Wu Y., Yang Y., Deng W., Ding X., Yang F., Xie C. (2022). The emergence of a multidrug-resistant and pathogenic ST42 lineage of staphylococcus haemolyticus from a hospital in China. Microbiol. Spectr..

[13] Nakakido M., Aikawa C., Nakagawa I., Tsumoto K. (2014). The staphylococcal elastin-binding protein regulates zinc-dependent growth/biofilm formation. J. Biochem..

[14] Harris L.G., Murray S., Pascoe B., Bray J., Meric G., Mageiros L., Wilkinson T.S., Jeeves R., Rohde H., Schwarz S. (2016). Biofilm morphotypes and population structure among staphylococcus epidermidis from commensal and clinical samples. PLoS ONE.

[15] Shibuya R., Uehara Y., Baba T., Teruya K., Satou K., Hirano T., Kirikae T., Hiramatsu K. (2020). Complete genome sequence of a methicillin-resistant staphylococcus lugdunensis strain and characteristics of its staphylococcal cassette chromosome mec. Sci. Rep..

[16] Jeon J., D’Souza R., Hong S.K., Lee Y., Yong D., Choi J., Lee K., Chong Y. (2014). Complete genome sequence of the siphoviral bacteriophage ymc/09/04/r1988 mrsa bp: A lytic phage from a methicillin-resistant staphylococcus aureus isolate. FEMS Microbiol. Lett..

[17] Chen H.-J., Tsai J.-C., Hung W.-C., Tseng S.-P., Hsueh P.-R., Teng L.-J. (2011). Identification of fusB-mediated fusidic acid resistance islands in staphylococcus epidermidis isolates. Antimicrob. Agents Chemother..

[18] Lacey K.A., Mulcahy M.E., Towell A.M., Geoghegan J.A., McLoughlin R.M. (2019). Clumping factor B is an important virulence factor during *Staphylococcus aureus* skin infection and a promising vaccine target. PLOS Pathog..

[19] Oliveira D.C., de Lencastre H. (2002). Multiplex pcr strategy for rapid identification of structural types and variants of the mec element in methicillin-resistant staphylococcus aureus. Antimicrob. Agents Chemother..

[20] McManus B.A., Coleman D.C., Deasy E.C., Brennan G.I., O’Connell B., Monecke S., Ehricht R., Leggett B., Leonard N., Shore A.C. (2015). Comparative genotypes, staphylococcal cassette chromosome mec (sccmec) genes and antimicrobial resistance amongst staphylococcus epidermidis and staphylococcus haemolyticus isolates from infections in humans and companion animals. PLoS ONE.

[21] Hosseinkhani F., Tammes Buirs M., Jabalameli F., Emaneini M., van Leeuwen W.B. (2018). High diversity in sccmec elements among multidrug-resistant staphylococcus haemolyticus strains originating from paediatric patients; characterization of a new composite island. J. Med. Microbiol..

[22] Chang S.-C., Lin L.-C., Lu J.-J. (2021). Comparative genomic analyses reveal potential factors responsible for the ST6 oxacillin-resistant *Staphylococcus lugdunensis* endemic in a hospital. Front. Microbiol..

[23] Liu J., Chen D., Peters B.M., Li L., Li B., Xu Z., Shirliff M.E. (2016). Staphylococcal chromosomal cassettes mec (SCCmec): A mobile genetic element in methicillin-resistant *Staphylococcus aureus*. Microb. Pathog..

[24] Harmer C.J., Hall R.M. (2019). An analysis of the is6/is26 family of insertion sequences: Is it a single family?. Microb. Genom..

[25] Fernandes P. (2016). Fusidic acid: A bacterial elongation factor inhibitor for the oral treatment of acute and chronic staphylococcal infections. Cold Spring Harb. Perspect. Med..

[26] Williamson D.A., Carter G.P., Howden B.P. (2017). Current and emerging topical antibacterials and antiseptics: Agents, action, and resistance patterns. Clin. Microbiol. Rev..

[27] Castanheira M., Watters A.A., Mendes R.E., Farrell D.J., Jones R.N. (2010). Occurrence and molecular characterization of fusidic acid resistance mechanisms among *Staphylococcus* spp. from European countries (2008). J. Antimicrob. Chemother..

[28] Hung W.-C., Chen H.-J., Lin Y.-T., Tsai J.-C., Chen C.-W., Lu H.-H., Tseng S.-P., Jheng Y.-Y., Leong K.H., Teng L.-J. (2015). Skin commensal staphylococci may act as reservoir for fusidic acid resistance genes. PLoS ONE.

[29] Yazdankhah S.P., Åsli A.W., Sørum H., Oppegaard H., Sunde M. (2006). Fusidic acid resistance, mediated by fusB, in bovine coagulase-negative staphylococci. J. Antimicrob. Chemother..

[30] Chen H.J., Hung W.C., Tseng S.P., Tsai J.C., Hsueh P.R., Teng L.J. (2010). Fusidic acid resistance determinants in staphylococcus aureus clinical isolates. Antimicrob. Agents Chemother..

[31] Castanheira M., Watters A.A., Bell J.M., Turnidge J.D., Jones R.N. (2010). Fusidic acid resistance rates and prevalence of resistance mechanisms among staphylococcus spp. Isolated in north America and Australia, 2007–2008. Antimicrob. Agents Chemother..

[32] Lina G., Quaglia A., Reverdy M.-E., Leclercq R., Vandenesch F., Etienne J. (1999). Distribution of genes encoding resistance to macrolides, lincosamides, and streptogramins among staphylococci. Antimicrob. Agents Chemother..

[33] Gatermann S.G., Koschinski T., Friedrich S. (2007). Distribution and expression of macrolide resistance genes in coagulase-negative staphylococci. Clin. Microbiol. Infect..

[34] Moxon E.R., Kroll J.S. (1990). The role of bacterial polysaccharide capsules as virulence factors. Curr. Top Microbiol. Immunol..

[35] Taylor C.M., Roberts I.S. (2005). Capsular polysaccharides and their role in virulence. Contrib. Microbiol..

[36] O’Riordan K., Lee J.C. (2004). *Staphylococcus aureus* capsular polysaccharides. Clin. Microbiol. Rev..

[37] Thammavongsa V., Kim H.K., Missiakas D., Schneewind O. (2015). Staphylococcal manipulation of host immune responses. Nat. Rev. Microbiol..

[38] Paharik A.E., Horswill A.R. (2016). The staphylococcal biofilm: Adhesins, regulation, and host response. Microbiol. Spectr..

[39] Entenza J.M., Foster T.J., Ni Eidhin D., Vaudaux P., Francioli P., Moreillon P. (2000). Contribution of clumping factor B to pathogenesis of experimental endocarditis due to *Staphylococcus aureus*. Infect. Immun..

[40] Panda S., Jena S., Sharma S., Dhawan B., Nath G., Singh D.V. (2016). Identification of novel sequence types among staphylococcus haemolyticus isolated from variety of infections in India. PLoS ONE.

[41] Kolmogorov M., Yuan J., Lin Y., Pevzner P.A. (2019). Assembly of long, error-prone reads using repeat graphs. Nat. Biotechnol..

[42] Boetzer M., Pirovano W. (2014). Sspace-longread: Scaffolding bacterial draft genomes using long read sequence information. BMC Bioinform..

[43] Gurevich A., Saveliev V., Vyahhi N., Tesler G. (2013). Quast: Quality assessment tool for genome assemblies. Bioinformatics.

[44] Wayne P. (2022). Performance Standards for Antimicrobial Susceptibility Testing.

[45] Warsa U.C., Nonoyama M., Ida T., Okamoto R., Okubo T., Shimauchi C., Kuga A., Inoue M. (1996). Detection of tet(K) and tet(M) in staphylococcus aureus of Asian countries by the polymerase chain reaction. J. Antibiot..

[46] Manoharan M., Sistla S., Ray P. (2021). Prevalence and molecular determinants of antimicrobial resistance in clinical isolates of *Staphylococcus haemolyticus* from India. Microb. Drug Resist..

[47] Chang S.C., Hidrosollo J.H., Lin L.C., Ou Y.H., Kao C.Y., Lu J.J. (2022). Characterization of oxacillin-resistant staphylococcus lugdunensis isolated from sterile body fluids in a medical center in Taiwan: A 12-year longitudinal epidemiological study. J. Microbiol. Immunol. Infect..

[48] Chen H.J., Chang Y.C., Tsai J.C., Hung W.C., Lin Y.T., You S.J., Tseng S.P., Teng L.J. (2013). New structure of phage-related islands carrying fusb and a virulence gene in fusidic acid-resistant staphy-lococcus epidermidis. Antimicrob. Agents Chemother..

