# Supplementary material for: Clonal Spreading of ST42 Staphylococcus haemolyticus Strains Occurs Possibly Due to fusB and tetK Resistant Genes and Capsule-Related Genes
Source: Int J Mol Sci. 2023 Mar 24;24(7):6198. doi: 10.3390/ijms24076198 (PMC10094739; doi:10.3390/ijms24076198)
Supplement: Supplementary file 1 [file ijms-24-06198-s001.zip › ijms-2240374-supplementary.pdf]

Clonal spreading of ST42 *Staphylococcus haemolyticus* strains occurs possibly due to *fusB* and *tetK* resistant genes and capsule-related genes

Supplementary

Figure S1. Numbers of *S. haemolyticus* ST3 and ST42 strains isolated from blood culture in Chang Hung Memorial Hospital from 2010 to 2017

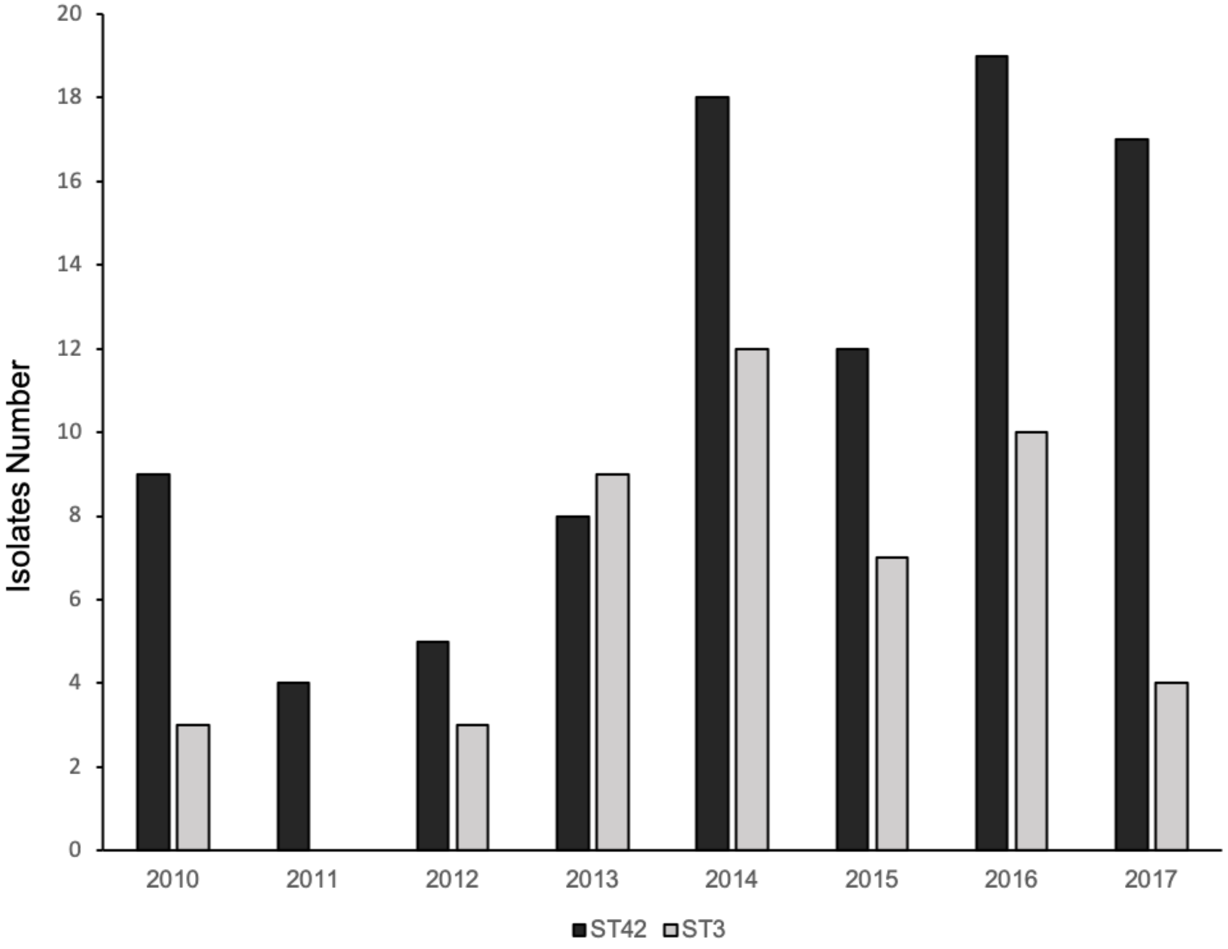

The preference distribution of 140 blood-isolated *S. haemolyticus* ST3 (48 isolates) and ST42 (92 isolates) was measured from 2010 to 2017. All isolates were collected from either Linkou or Kaohsiung Chang Gung Memorial Hospital, and several isolates have been published in previous reports.

Figure S2. Correlation of fusidic acid and tetracycline topic usage amount and bacterial resistant strain proportion in Linkou Chang Hung Memorial Hospital from 2011 to 2017

A. Fusidic acid topic usage and fusidic acid resistant isolates proportion distribution from 2011 to 2017

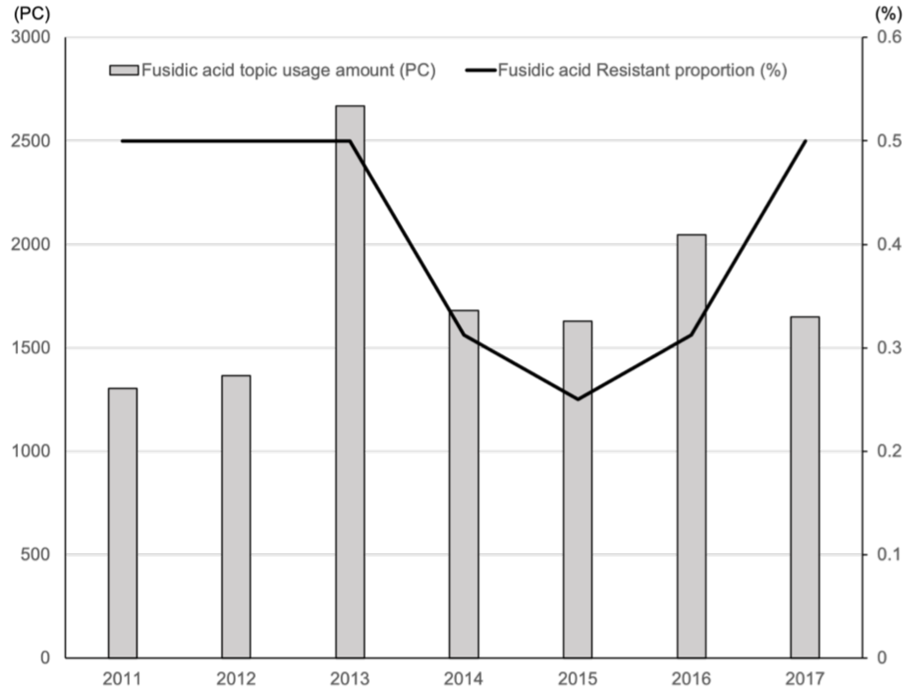

B. Tetracycline topic usage amount and tetracycline resistant isolates proportion distribution from 2011 to 2017

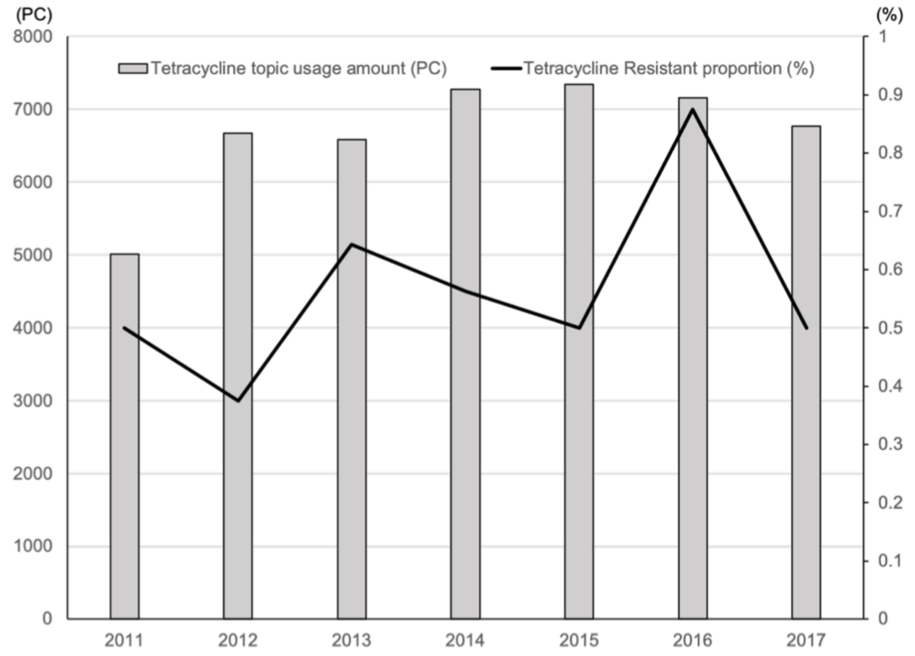

The fusidic acid and tetracycline topic usage amount and their resistant isolates were investigated from Linkou Chang Gung Memorial hospital during 2011 to 2017. Drug usage data were only count for

hospitalized usage amount. Correlation of these two drugs with their resistant isolates' distribution proportion were measured by correlation coefficient; -0.01 for fusidic acid and its resistant isolates, and 0.26 for tetracycline and its resistant isolates.

Table S1 Information of 140 *S. haemolyticus* isolates used in this study

Isolated Origin	Collected Year	MLST	Drug Resistant Phenotype*					Drug Resistant Genes					
			CC	E	FA	TE	C	*mph(C)*	*msrA*	*ermC*	*fusB*	*tet(K)*	*cat*
Taipei	2010	3	R	R	S	S	S	+	+	+	-	-	-
Taipei	2010	3	R	R	S	S	R	-	-	+	-	-	+
Taipei	2010	3	S	R	R	S	S	+	+	+	+	-	-
Taipei	2010	42	R	R	R	R	S	+	+	+	+	+	-
Taipei	2010	42	R	R	R	R	S	+	+	+	+	+	-
Taipei	2010	42	R	R	R	R	S	+	+	+	+	+	-
Taipei	2010	42	R	R	R	R	S	+	+	+	+	+	-
Taipei	2010	42	R	R	R	R	S	+	+	+	+	+	-
Taipei	2010	42	R	R	R	S	S	+	+	+	+	-	-
Taipei	2010	42	R	R	R	R	S	+	+	+	+	+	-
Taipei	2010	42	R	R	R	R	S	+	+	+	+	+	-
Taipei	2010	42	S	R	R	R	S	+	+	-	+	+	-
Taipei	2011	42	R	R	S	S	S	+	+	+	-	-	-
Taipei	2011	42	R	R	R	R	S	+	+	+	+	+	-
Taipei	2011	42	R	R	S	S	S	+	+	-	-	-	-
Taipei	2011	42	R	R	R	R	S	+	+	+	+	+	-
Taipei	2012	3	R	R	S	S	R	-	-	+	-	-	+
Taipei	2012	3	R	R	S	S	S	+	+	+	-	-	-
Taipei	2012	3	R	R	S	S	R	-	-	+	-	-	+
Taipei	2012	42	R	R	R	S	S	+	+	+	+	-	-
Taipei	2012	42	R	R	R	R	S	+	+	+	+	+	-
Taipei	2012	42	S	R	R	R	S	+	+	+	+	+	-
Taipei	2012	42	S	R	R	R	S	+	+	+	+	+	-
Taipei	2012	42	R	R	S	S	S	+	+	+	-	-	-
Taipei	2013	3	R	R	S	S	S	+	+	+	-	-	-
Taipei	2013	3	R	R	S	S	R	-	-	+	-	-	+
Taipei	2013	3	R	R	R	S	S	+	+	+	+	-	-
Taipei	2013	3	R	R	S	S	S	-	-	+	-	-	-
Taipei	2013	3	R	R	R	S	S	+	+	+	+	-	-
Taipei	2013	3	S	R	S	R	S	-	-	+	-	+	-
Taipei	2013	42	R	R	S	R	S	+	+	+	-	+	-
Taipei	2013	42	R	R	R	R	S	+	+	+	+	+	-

Taipei	2013	42	S	R	R	R	S	+	+	+	+	+	-
Taipei	2013	42	R	R	R	R	S	+	+	+	+	+	-
Taipei	2013	42	S	R	R	R	S	+	+	-	+	+	-
Taipei	2013	42	R	R	S	R	S	+	+	+	-	+	-
Taipei	2013	42	S	R	R	R	S	+	+	+	+	+	-
Taipei	2013	42	S	R	S	R	S	+	+	+	-	+	-
Kaohsiung	2013	3	S	R	S	S	S	+	+	+	-	-	-
Kaohsiung	2013	3	S	R	S	S	S	+	+	+	-	-	-
Kaohsiung	2013	3	R	R	S	S	S	-	-	+	-	-	-
Taipei	2014	3	R	R	S	S	S	-	-	+	-	-	-
Taipei	2014	3	S	R	S	S	S	+	+	+	-	-	-
Taipei	2014	3	R	R	S	S	S	+	+	+	-	-	-
Taipei	2014	3	R	R	R	S	S	+	+	+	+	-	-
Taipei	2014	3	R	R	S	S	S	+	+	+	-	-	-
Taipei	2014	3	R	R	S	S	S	-	-	+	-	-	-
Taipei	2014	42	R	R	R	R	S	+	+	+	+	+	-
Taipei	2014	42	S	R	R	R	S	+	+	+	+	+	-
Taipei	2014	42	S	R	S	R	S	+	+	+	-	+	-
Taipei	2014	42	R	R	R	R	R	+	+	+	+	+	+
Taipei	2014	42	S	R	S	R	S	+	+	+	-	+	-
Taipei	2014	42	S	R	R	R	S	+	+	-	+	+	-
Taipei	2014	42	R	R	S	R	S	+	+	+	-	+	-
Taipei	2014	42	R	R	S	R	S	+	+	+	-	+	-
Taipei	2014	42	R	R	S	S	S	+	+	-	-	-	-
Taipei	2014	42	R	R	S	R	S	+	+	+	-	+	-
Kaohsiung	2014	3	S	R	S	S	S	+	+	+	-	-	-
Kaohsiung	2014	3	S	R	S	R	S	+	+	-	-	+	-
Kaohsiung	2014	3	S	R	S	S	S	+	+	-	-	-	-
Kaohsiung	2014	3	R	R	S	S	R	-	-	-	-	-	+
Kaohsiung	2014	3	S	R	S	S	S	+	+	+	-	-	-
Kaohsiung	2014	3	S	R	S	S	S	+	+	+	-	-	-
Kaohsiung	2014	42	R	R	R	R	S	+	+	+	+	+	-
Kaohsiung	2014	42	R	R	S	S	S	+	+	+	-	-	-
Kaohsiung	2014	42	S	R	S	R	S	+	+	+	-	+	-
Kaohsiung	2014	42	R	R	S	S	S	+	+	+	-	-	-
Kaohsiung	2014	42	S	R	S	R	S	+	+	-	-	+	-

Kaohsiung	2014	42	S	R	R	S	S	-	-	+	-	-	-
Kaohsiung	2014	42	S	R	S	R	S	+	+	+	-	+	-
Kaohsiung	2014	42	R	R	S	R	S	+	+	+	-	+	-
Taipei	2015	3	R	R	S	S	R	-	-	+	-	-	+
Taipei	2015	3	R	R	S	S	S	+	+	+	-	-	-
Taipei	2015	3	R	R	S	S	S	+	+	+	-	-	-
Taipei	2015	42	R	R	S	S	S	+	+	+	-	-	-
Taipei	2015	42	S	R	S	R	S	+	+	+	-	+	-
Taipei	2015	42	S	R	R	R	S	+	+	+	+	+	-
Taipei	2015	42	R	R	R	R	S	+	+	+	+	+	-
Taipei	2015	42	R	R	S	R	S	+	+	+	-	+	-
Kaohsiung	2015	3	S	R	S	S	S	+	+	-	-	-	-
Kaohsiung	2015	3	R	R	S	S	S	+	+	+	-	-	-
Kaohsiung	2015	3	R	R	S	S	S	+	+	+	-	-	-
Kaohsiung	2015	3	R	R	R	S	S	+	+	+	+	-	-
Kaohsiung	2015	42	R	R	R	R	S	+	+	+	+	+	-
Kaohsiung	2015	42	R	R	S	S	S	+	+	-	-	-	-
Kaohsiung	2015	42	R	R	S	R	S	+	+	+	-	+	-
Kaohsiung	2015	42	S	R	S	S	R	+	+	+	-	-	+
Kaohsiung	2015	42	S	R	S	S	S	+	+	+	-	-	-
Kaohsiung	2015	42	S	R	R	R	S	+	+	+	+	+	-
Kaohsiung	2015	42	R	R	S	S	S	+	+	+	-	-	-
Taipei	2016	3	R	R	S	S	R	-	-	+	-	-	+
Taipei	2016	3	R	R	S	S	S	+	+	+	-	-	-
Taipei	2016	42	R	R	S	R	S	+	+	+	-	+	-
Taipei	2016	42	S	R	S	R	S	+	+	+	-	+	-
Taipei	2016	42	S	R	S	R	S	+	+	+	-	+	-
Taipei	2016	42	S	R	R	R	S	+	+	+	+	+	-
Taipei	2016	42	S	R	S	R	S	+	+	+	-	+	-
Taipei	2016	42	R	R	R	R	S	+	+	+	+	+	-
Taipei	2016	42	R	R	S	R	S	+	+	+	-	+	-
Taipei	2016	42	S	R	S	R	S	+	+	-	-	+	-
Taipei	2016	42	R	R	S	R	S	+	+	+	-	+	-
Taipei	2016	42	S	R	R	R	S	+	+	-	+	+	-
Taipei	2016	42	S	R	S	R	S	+	+	+	-	+	-
Taipei	2016	42	R	R	R	S	S	+	+	+	+	-	-

Taipei	2016	42	S	R	R	R	S	+	+	+	+	+	-
Taipei	2016	42	R	R	S	R	S	+	+	+	-	+	-
Kaohsiung	2016	3	S	R	S	S	S	+	+	+	-	-	-
Kaohsiung	2016	3	S	R	R	S	S	+	+	+	+	-	-
Kaohsiung	2016	3	S	R	R	S	S	+	+	+	+	-	-
Kaohsiung	2016	3	S	R	R	S	S	+	+	+	+	-	-
Kaohsiung	2016	3	R	R	S	S	S	+	+	+	-	-	-
Kaohsiung	2016	3	R	R	R	S	S	+	+	+	+	-	-
Kaohsiung	2016	3	R	R	S	S	S	+	+	+	-	-	-
Kaohsiung	2016	3	S	R	R	S	S	+	+	-	+	-	-
Kaohsiung	2016	42	R	R	S	R	S	+	+	+	-	+	-
Kaohsiung	2016	42	R	R	S	S	R	+	+	+	-	-	+
Kaohsiung	2016	42	R	R	S	S	S	+	+	+	-	-	-
Kaohsiung	2016	42	R	R	S	S	R	+	+	+	-	-	+
Kaohsiung	2016	42	S	R	S	S	S	+	+	+	-	-	-
Taipei	2017	3	R	R	S	S	S	+	+	+	-	-	-
Taipei	2017	3	R	R	S	S	S	+	+	+	-	-	-
Taipei	2017	42	R	R	R	S	S	+	+	+	+	-	-
Taipei	2017	42	R	R	R	S	S	+	+	+	+	-	-
Taipei	2017	42	S	R	S	R	S	+	+	+	-	+	-
Taipei	2017	42	S	R	R	R	S	+	+	+	+	+	-
Taipei	2017	42	R	R	S	R	S	+	+	+	-	+	-
Taipei	2017	42	S	R	S	S	S	+	+	+	-	-	-
Taipei	2017	42	S	R	R	R	S	+	+	+	+	+	-
Taipei	2017	42	S	R	R	R	S	+	+	-	+	+	-
Taipei	2017	42	S	R	S	R	S	+	+	-	-	+	-
Taipei	2017	42	R	R	R	S	S	+	+	+	+	-	-
Taipei	2017	42	S	R	S	R	S	+	+	+	-	+	-
Taipei	2017	42	R	R	R	S	S	+	+	+	+	-	-
Kaohsiung	2017	3	R	R	S	S	S	+	+	+	-	-	-
Kaohsiung	2017	3	R	R	R	S	S	+	+	+	+	-	-
Kaohsiung	2017	42	R	R	S	R	S	+	+	+	-	+	-
Kaohsiung	2017	42	R	R	R	S	S	+	+	+	+	-	-
Kaohsiung	2017	42	R	R	S	R	S	+	+	+	-	+	-
Kaohsiung	2017	42	R	R	S	S	S	+	+	+	-	-	-
Kaohsiung	2017	42	R	R	S	S	S	+	+	+	-	-	-

* Abbreviation of five antibiotics used for strain drug susceptibilities assay: CC, clindamycin; E, erythromycin; FA, fusidic acid; TE, tetracycline; C, chloramphenicol. All 140 isolates were resistant to oxacillin and penicillin.

Isolates containing both *fusB* and *tetK* genes are indicated by red color and bold font.
